# Commentary: Type I CRISPR-Cas targets endogenous genes and regulates virulence to evade mammalian host immunity

**DOI:** 10.3389/fmicb.2017.00319

**Published:** 2017-02-28

**Authors:** Hanna Müller-Esparza, Lennart Randau

**Affiliations:** Prokaryotic Small RNA Biology Group, Max Planck Institute for Terrestrial MicrobiologyMarburg, Germany

**Keywords:** CRISPR, Cas proteins, Cascade, ribonucleoprotein, RNA interference

Type-I CRISPR-Cas systems are abundant antiviral defense systems of bacteria and archaea. The hallmark sequences of these systems are short CRISPR RNAs (crRNAs) that contain spacer sequences which guide an interference complex termed Cascade (CRISPR-associated complex for antiviral defense) toward their viral DNA target (van der Oost et al., 2014). In the recent years, several subtypes of this Type I CRISPR-Cas system have been studied in detail and all interference complexes share a central crRNA whose spacer is protected by a multi-subunit filament of Cas7 (Csy3) backbone proteins (Gleditzsch et al., 2016). The termini of the CRISPR RNA are capped by Cas5 (Csy2) and Cas6 proteins and additional large and small subunits can mediate interactions with the target DNA, i.e., the protospacer and the protospacer-adjacent motif (PAM). The crRNA spacer is used to scan DNA for complementary bases and the large subunit identifies PAM sequences to achieve self- versus non-self-discrimination and to avoid self-targeting. Finally, Cascade complexes recruit a helicase/nuclease, termed Cas3, to degrade identified DNAs (Hochstrasser et al., 2014).

In a recent study in Cell Research (Li et al., 2016), Li et al., highlight a surprising deviation from these established concepts and show self-targeting of the lasR mRNA based on only nine nucleotide complementarity between the CRISPR RNA and the target mRNA, as well as the presence of a small “5′-GGN-3′” recognition motif. Earlier studies have indicated that *Escherichia coli* Type I-E Cascade can bind ssRNA *in vitro* (Jore et al., 2011) and that Cas3 can degrade ssRNA (Beloglazova et al., 2011). However, specific DNA targeting is considered to be essential for proper identification of foreign DNA elements while maintaining genome integrity. The described RNA targeting relies on a discrimination mechanism similar to the established DNA targeting pathway and suggests that a 5′-GGN-3′ PAM-like sequence must exist to mark the mRNA-target. However, a conventional PAM sequence in Type I-F Cascade prevents base pairing between the 8 nt 5′-repeat-tag of the crRNA and the DNA target (Rollins et al., 2015), while the identified PAM-like sequence in the lasR mRNA would prevent base pairing with the 3′-tag (Figure 1A). This implies that Cascade would have evolved a specific RNA target discrimination mechanism. The 3′-tag of a crRNA is bound by Cas6f in Type I-F systems. Thus, this protein would be in the vicinity of the PAM-like sequence, while Cas proteins involved in conventional PAM recognition are located at the opposite end of the Cascade complex (Figure 1A). Surprisingly, a very short 9 bp sequence downstream of the PAM-like sequence was sufficient for *in vitro* RNA cleavage by recombinant Cascade. Here, all nucleotides of this region were important, even though every 6th nucleotide is splayed out in the Type I-E Cascade structure at the Cas7 backbone filament junctions and was found not to be involved in base pairing with the target (Wiedenheft et al., 2011). Thus, both PAM-like sequence and mRNA target recognition clearly deviate from the mechanisms established for conventional DNA recognition by Cascade.

**Figure 1:**
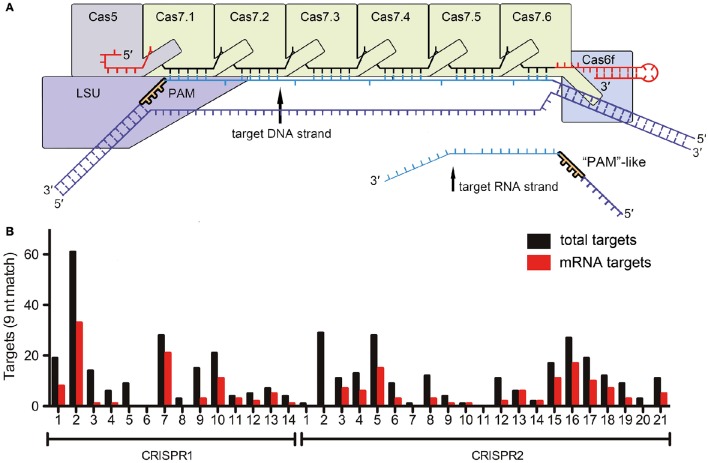
**RNA targeting by Type I-F Cascade (A)** Schematic representation of a Type I-F Cascade complex with bound DNA. The approximate location of the PAM near the large subunit (LSU, Csy1) is indicated. The reported target mRNA would bind at the opposite end of Cascade (near Cas7.6 and Cas6f). **(B)** The number of targets for each *Pseudomonas aeruginosa* strain UCBPP-PA14 spacer (5′-GGN-3′ followed by nine base-pair complementarity) in the genome (total targets) and transcriptome (mRNA targets) is indicated.

The authors did not investigate the impact of this non-stringent mRNA targeting mechanism on the global bacterial RNA metabolism. Therefore, we screened all CRISPR spacers of the two CRISPR systems of *Pseudomonas aeruginosa* strain UCBPP-PA14 for possible crRNA-mRNA interactions. A 5′-GGN-3′ sequence, followed by 12 or 9 bp complementarity, were considered to be requirements for potential interactions. In total, 11 genomic targets were obtained for 12 nt spacer matches. Five of these hits are located on mRNAs, while only one hit is located in a non-coding region. Next, we reduced the number of required continuous base pairs to nine nucleotides as suggested for the described Type I-F Cascade *in vitro* lasR mRNA decay activity. With these parameters, the crRNAs would potentially target 422 host genome regions, of which 31 (7.3%) are located in intergenic regions and 391 (92.7%) are present in coding regions (Figure 1B). The sense strand (i.e., the mRNA) is targeted for 189 of the 391 genes (48.3%). In conclusion, there does not appear to be a selection against mRNA targeting. The existence of this large number of potential targets suggests two possible scenarios. First, CRISPR-Cas systems could play a major role in regulating the abundance of individual mRNAs and shape bacterial transcriptomes. Alternatively, the observed RNA targeting of the lasR mRNA might be an exceptional occurrence and cells would need to have evolved protection mechanisms against off-target mRNA degradation. A global analysis of transcriptome changes upon CRISPR-Cas induction and/or deletion would be necessary to be able to differentiate between these possibilities.

## Author contributions

LR wrote the manuscript. LR and HM analyzed crRNA targets.

## Funding

Funding by the DFG (FOR1680) is acknowledged.

### Conflict of interest statement

The authors declare that the research was conducted in the absence of any commercial or financial relationships that could be construed as a potential conflict of interest.

## References

[B1] Beloglazova N. Petit P. Flick R. Brown G. Savchenko A. Yakunin A. F. (2011). Structure and activity of the Cas3 HD nuclease MJ0384, an effector enzyme of the CRISPR interference. EMBO J. 30, 4616–4627. 10.1038/emboj.2011.377 22009198 PMC3243599

[B2] Gleditzsch D. Muller-Esparza H. Pausch P. Sharma K. Dwarakanath S. Urlaub H.. (2016). Modulating the Cascade architecture of a minimal Type I-F CRISPR-Cas system. Nucleic Acids Res. 44, 5872–5882. 10.1093/nar/gkw469 27216815 PMC4937334

[B3] Hochstrasser M. L. Taylor D. W. Bhat P. Guegler C. K. Sternberg S. H. Nogales E.. (2014). CasA mediates Cas3-catalyzed target degradation during CRISPR RNA-guided interference. Proc. Natl. Acad. Sci. U.S.A. 111, 6618–6623. 10.1073/pnas.1405079111 24748111 PMC4020112

[B4] Jore M. M. Lundgren M. van Duijn E. Bultema J. B. Westra E. R. Waghmare S. P.. (2011). Structural basis for CRISPR RNA-guided DNA recognition by Cascade. Nat. Struct. Mol. Biol. 18, 529–536. 10.1038/nsmb.2019 21460843

[B5] Li R. Fang L. Tan S. Yu M. Li X. He S.. (2016). Type I CRISPR-Cas targets endogenous genes and regulates virulence to evade mammalian host immunity. Cell Res. 26, 1273–1287. 10.1038/cr.2016.135 27857054 PMC5143421

[B6] Rollins M. F. Schuman J. T. Paulus K. Bukhari H. S. Wiedenheft B. (2015). Mechanism of foreign DNA recognition by a CRISPR RNA-guided surveillance complex from *Pseudomonas aeruginosa*. Nucleic Acids Res. 43, 2216–2222. 10.1093/nar/gkv094 25662606 PMC4344526

[B7] van der Oost J. Westra E. R. Jackson R. N. Wiedenheft B. (2014). Unravelling the structural and mechanistic basis of CRISPR-Cas systems. Nat. Rev. Microbiol. 12, 479–492. 10.1038/nrmicro3279 24909109 PMC4225775

[B8] Wiedenheft B. Lander G. C. Zhou K. Jore M. M. Brouns S. J. van der Oost J.. (2011). Structures of the RNA-guided surveillance complex from a bacterial immune system. Nature 477, 486–489. 10.1038/nature10402 21938068 PMC4165517

